# Single-cell Hi-C data analysis: safety in numbers

**DOI:** 10.1093/bib/bbab316

**Published:** 2021-08-18

**Authors:** Aleksandra A Galitsyna, Mikhail S Gelfand

**Affiliations:** Skolkovo Institute of Science and Technology, Skolkovo, Russia; Institute for Information Transmission Problems, RAS, Moscow, Russia; Institute of Gene Biology, RAS, Moscow, Russia; Skolkovo Institute of Science and Technology, Skolkovo, Russia; Institute for Information Transmission Problems, RAS, Moscow, Russia

**Keywords:** single cell, chromatin, single-cell Hi-C, conformation capture, single-cell sequencing

## Abstract

Over the past decade, genome-wide assays for chromatin interactions in single cells have enabled the study of individual nuclei at unprecedented resolution and throughput. Current chromosome conformation capture techniques survey contacts for up to tens of thousands of individual cells, improving our understanding of genome function in 3D. However, these methods recover a small fraction of all contacts in single cells, requiring specialised processing of sparse interactome data. In this review, we highlight recent advances in methods for the interpretation of single-cell genomic contacts. After discussing the strengths and limitations of these methods, we outline frontiers for future development in this rapidly moving field.

## Introduction

Detecting specific DNA positioning in single cells was first proposed over half a century ago [44, 72]. Deriving statistically reliable general patterns of chromatin folding in single cells, however, has been challenging [5]. Improvements towards this goal have included: increasing the number of analysed cells, studying more loci (up to the complete genome), reducing the size of the interacting regions and improving discriminative power for detection of contacts at a broader scale of spatial distances. There are two main approaches: *microscopy based* and *capture based*. These two types of methods, despite their limitations, provide complementary views on the chromatin structure of single cells [92].

*Targeted microscopy* approaches measure spatial distances between genomic regions in individual cells using labelled probes. These typically involve complicated probe design, which can be overcome with a new *in situsequencing* technique [73] but remains challenging to implement. With any microscopy approach, trade-offs have to be considered: which cells are analysed (fixed or living), number of targeted regions, time dynamics and resolution of obtained images. For an extended discussion, we refer the reader to recent reviews [5, 8].

*Chromosome conformation capture* uses crosslinking, digestion and proximity ligation to detect genomic regions located close to each other in 3D space. It was originally designed for inputs of millions of cells and had higher statistical power than microscopy [23]. An explosion of conformation-based techniques, including the high-throughput sequencing-based Hi-C [64], has paved the way for new discoveries expanding our general understanding of DNA folding in eukaryotic cells [34], bacterial cells [19] and even viruses [9]. For eukaryotes, these patterns include *topologically associating domains (TADs)*, promoter-enhancer and architectural *loops* and *compartments* (reviewed in-depth by [6, 21, 22, 84]).

A long-standing impediment to our interpretation and understanding of structure formation principles is that chromatin features in individual cells are not equivalent to the average features in a population of cells [31]. To address this problem, the first *single-cell chromosome conformation capture assay (scHi-C)* reduced the scale of the traditional Hi-C protocol to one cell per reaction tube [68]. Then, scHi-C was extended by introduction of sorting into multi-well plates and tagmentation followed by polymerase chain reaction (PCR) [69]. A similar approach, *single-nucleus Hi-C (snHi-C)* substituted traditional PCR with whole-genome amplification and cut out the biotin fill-in step. This came, however, at the cost of larger sequencing volumes and data processing [28, 32]. *Diploid chromatin conformation capture (Dip-C)* has adapted tagmentation-based strategies [86, 87], simplifying the experimental protocol [85]. Single-cell combinatorial indexed Hi-C (sciHi-C) is yet another powerful technique based on several rounds of combinatorial barcoding of diluted samples without isolation of individual cells [49, 76]. scHi-C can be combined with other assays to investigate the methylome, such as *Methyl-3C* and *sn-m3C-seq* [55, 57]. For the sake of simplicity, we will refer to all the family of methods a scHi-C throughout this review.

Alongside scHi-C, there is a growing family of many-body interaction capture methods, including *MC-3C* [88], *PORE-C* [91], *Nano-C* [13]. These methods recover up to several dozens of pairwise contacts from individual cells but cannot yet compete with scHi-C in genome-wide searches for architectural features. *Single-cell SPRITE* is a ligation-free method that generates 30 times more contacts but captures interacting complexes instead of pairs [2].

The main challenge of analysing scHi-C data is extreme data sparsity. On average, up to 700 000 interactions are captured in any given cell (for mouse [55]). Thus, the power of scHi-C manifests itself when data for multiple cells are available. Firstly, it makes the detection of chromatin patterns of individual cells statistically reliable. Twenty cells may already be sufficient to assess the presence of TADs, compartments and loops at the level of individual cells of *Drosophila* [93]. Secondly, multiple cells may be clustered into groups of similar types and pooled *in silico*. Such pseudo-bulk Hi-C of scHi-C-guided groups is a better alternative to bulk Hi-C, where the contacts formed in different cell types are indistinguishable [69, 85, 87]. To analyse such data, one needs specialised tools and computational pipelines, which are currently designed *ad hoc* and are rarely re-used or cross-tested. Here, we describe the diversity of recent scHi-C studies and summarise computational approaches to single-cell interactome data (for a recent review of similar topics, see [102]).

## Overview of single-cell Hi-C techniques

**Figure 1:**
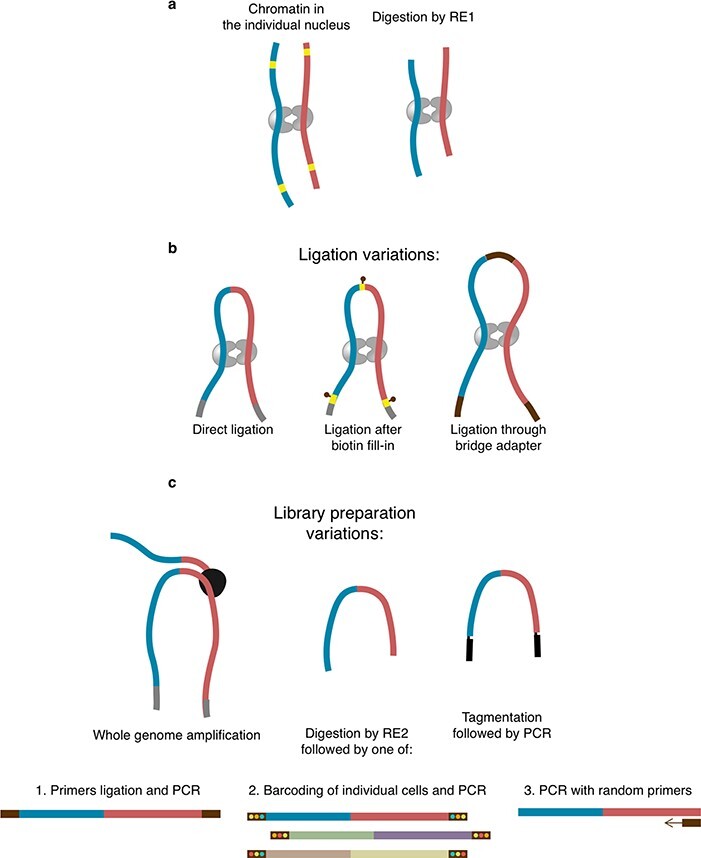
Overview of variations in scHi-C protocols relevant for data processing. **A**. Cross-linking and digestion, used in any scHi-C. **B**. Variations of the ligation step. **C**. Variations of the library preparation. RE1 and RE2 denote restriction enzymes selected for corresponding stages.

Like traditional bulk Hi-C, single-cell Hi-C includes chromatin crosslinking, cells permeabilisation, DNA digestion, proximity ligation and library preparation. A crucial step of scHi-C, however, is either *isolation* or *barcoding* of individual cells. To separate contacts from each nucleus, a typical approach is to isolate cells or nuclei into individual reaction mixtures and perform subsequent steps separately. The isolation can be done following crosslinking of cells [28, 82], after ligation [85–87] or right before de-crosslinking [16, 68, 69]. Technically, this is performed by manual placement of each nucleus into a single tube [28, 32] or fluorescence-activated cell/nucleus sorting (FACS/FANS) into individual wells of a plate [16, 82, 87]. Right before or during sorting, optional steps can be included, such as imaging [52, 82] or bisulfite conversion [55, 57]. Isolation-free technique *single-cell combinatorial indexed Hi-C (sciHi-C)* involves several rounds of combinatorial barcoding of the diluted cells [49, 76]. Isolation-free sciHi-C requires demultiplexing as one of the first data processing steps, while the isolation approach may [69] or may not include this step. A more comprehensive overview of the scHi-C experimental technique can be found [92], but we will highlight aspects of different protocols that are particularly relevant for data processing (Figure 1).

The initial step of the scHi-C protocol is to crosslink cells with formaldehyde, resulting in the fixation of DNA-DNA interactions. Next, cell membranes are lysed to guarantee the delivery of reagents into the nucleus. Then, DNA is digested by a restriction enzyme such as DpnII that cuts at the four-letter palindromic motif GATC (Figure 1A). This produces free ends of restriction fragments, which are then ligated either directly [28, 32, 85–87], after biotin fill-in [68, 69, 82] or after ligation of a biotinylated bridge adaptor [49, 76] (Figure 1B). Ligation junctions containing biotin-labelled nucleotides are pulled down using streptavidin. This pulldown is omitted in some scHi-C variants because it results in a loss of meaningful contacts [28, 32, 85–87]. Regardless of the ligation procedure, properly formed junctions are expected to contain specific sequences (restriction sites with or without a bridge, Figure 1), which can be used to computationally select real contacts [93]. The final step of scHi-C is to extract DNA and prepare it for sequencing. Multiple library preparation strategies were probed with scHi-C (Figure 1C), including whole-genome amplification (Illustra WGA in [28], META WGA in [87]), tagmentation followed by PCR [69], digestion with a restriction enzyme followed by primers ligation and PCR [68], barcoding and PCR [76] or PCR with random primers [57]. While tagmentation and restriction enzyme digestion generate fixed-point cuts in the DNA resulting in simple rules for computational deduplication of the pairs with coinciding mapping positions, this is not the case for whole-genome amplification and PCR with random primers, for which other deduplication schemes should be used. Finally, amplified DNA is purified and sequenced in the paired-end mode.

## Data processing workflow

**Figure 2:**
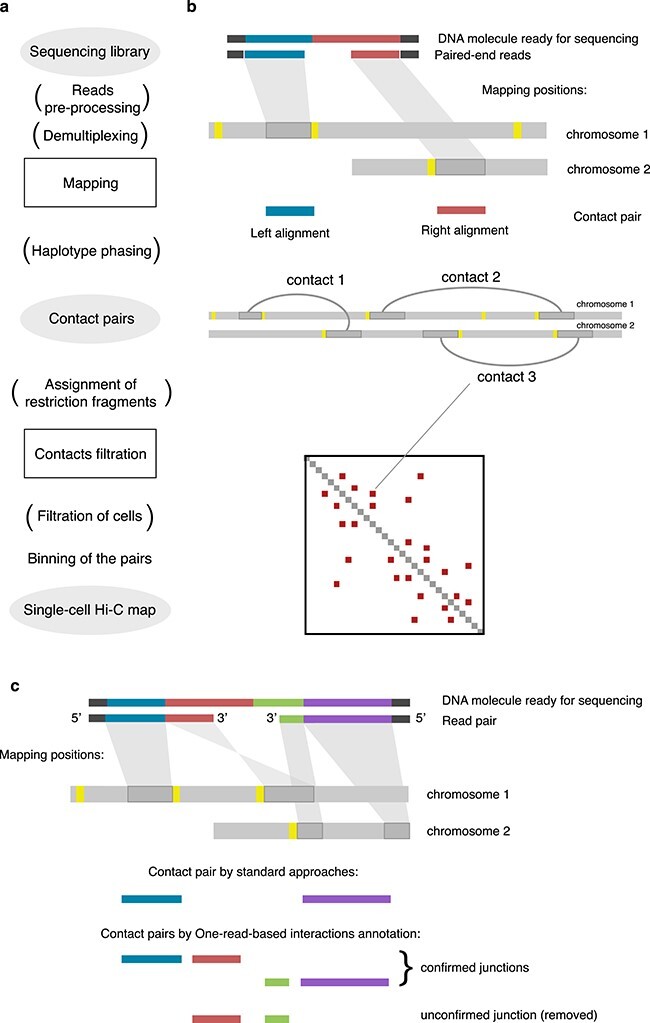
Outline of single-cell Hi-C data processing. The steps in brackets are optional, depending on the scHi-C protocol and the pipeline specifics.

The data processing workflow (Figure 2A and B) consists of general steps shared with typical Hi-C: optional pre-processing of reads (trimming, demultiplexing, etc.), read mapping, optional restriction fragment assignment, filtration of contacts and deduplication and binning with generation of single-cell Hi-C maps. The cells are typically filtered by the quality and/or the number of contacts.

### Mapping of reads

As with any other conformation capture, scHi-C generates chimeric DNA molecules (Figure 2C), making the mapping of these discontinuous reads to multiple genomic locations non-trivial [51]. Standard mappers, such as bowtie2 [54], cannot reliably map such reads. There are four main approaches to treat scHi-C chimaeras, three of them transferred from traditional bulk Hi-C: split read alignment, iterative mapping and read clipping. The fourth approach is one-read-based mapping (ORBITA), a special case of the split read alignment [93], which attempts to find only those contact pairs that are directly ligated (Figure 2c). In the *split read alignment* strategy, specialised mappers like bwa mem [58] detect multiple sequential alignments in each read. Of these, only the representative alignments are retained (typically, the alignments at 5$^\prime $-end). Some studies use the information about 3$^\prime $-end alignments to specify the endpoints of contacting fragments [86]. *Iterative mapping* is a method of analysing chimeric reads initially used for traditional Hi-C [42] and adapted for single-cells [28, 32]: short 5$^\prime $ sequences of increasing size are iteratively selected on both forward and reverse reads until the mapping of the pair (or coverage of the full read length) is achieved [51]. In *read clipping*, reads are scanned for the restriction site [69, 82] or bridge adapter [76], and all the 3$^\prime $ sequences after the match are removed. Two resulting paired sequences (one for forward and one for reverse read) are mapped independently and form a contact pair if the mapping was successful. However, only *one-read-based interactions annotation* utilises the information on chimeric parts to guarantees that the observed pair is a direct ligation junction of DNA fragments (Figure 2c). This approach reduces erroneous contacts in scHi-C data [93].

Another problem during scHi-C read mapping is genetic variation. Some regions of the genome of the studied cells differ from the reference hampering the mappability. Moreover, the cells are not guaranteed to descend from a single clone [86] and may have intrinsic variation, such as single-nucleotide polymorphisms (SNPs). Thus, some studies [82] ignore genomic locations with SNPs and prohibit mapping mismatches. On the other hand, SNPs can be a powerful source of information to help distinguish haplotype alleles [16, 69, 86] and impute the contacts of the maternal and paternal chromosomes [86].

### Filtering of contacts

After mapping, the scHi-C maps are vastly populated with *amplification duplicates, contacts of promiscuous genomic regions* and *artifactual contacts*, which can be detected and filtered out.

*Amplification duplicates* are identical or nearly identical copies of the same contact pairs generated during library preparation. Depending on the experimental protocol, the scHi-C duplicates do not necessarily have the same mapping positions in the genome. Whole-genome amplification and PCR with random primers produce DNA fragments that may originate at random locations close to actual ligation position. Thus, if a group of contact pairs has the same restriction fragments [76] or their termini [69, 93], these contacts are likely to have been duplicated and should be merged into a single contact. Alternatively, contacts of the same 500 bp-bins [28] or contacts located closer than 1 kb [86] may be merged directly [28] or iteratively [86].

The genome coverage in conformation capture is affected by multiple factors, including replication, DNA accessibility, GC-content and active chromatin state [42, 78, 97]. In bulk Hi-C, this is mitigated by correction, such as iterative balancing [42]. However, due to data sparsity, this step is not recommended for scHi-C (although proposed as intermediary step of quality assessment [40]), and little research has been devoted to scHi-C correction alternatives [59, 66]. In the absence of data correction, scHi-C may bear intrinsic biases, such as larger numbers of contacts formed by active regions [93] and early replication domains [69]. Larger numbers of contacts have been suggested for regions with genomic rearrangements [69], e.g. Stevens *et al.* [82] detected trisomy by the increased number of contacts for the whole chromosome. As a partial remedy, one can remove *contacts of promiscuous genomic regions* [68, 69, 93], e.g. 1 Kb regions that have more than ten contacts in a given cell [86].

*Artifactual contacts* are random contacts happening at various stages of scHi-C sample preparation and data processing, typically not representative of the real 3D conformation of chromatin and impairing downstream analysis. First of all, properly formed and mapped pairs should be located close to the restriction sites. scHi-C protocols using Phi29 phage polymerase can generate switch templates during WGA that are devoid of this feature and should be discarded [93]. The original scHi-C protocol generates a number of *spurious ligations*, likely represented by the pairs supported by a single read [68]. Frequent artefacts are *sequencing pairing mismatches*, having a global rate of $0.1\%$ for Illumina [69, 76], as assessed by admixture of phiX174 DNA to mouse cells [69]. Stevens *et al.* [82] suggested a general scheme for filtering a broad range of scHi-C artefacts, which is based on the assumption that the regions in close spatial proximity have the neighbouring genomic regions located nearby, also forming a contact. Thus, if the contact is isolated (e.g. is not supported by neighbours within 2 Mb distance [82]), it is likely to represent an artefact and should be removed [82, 86].

### Filtering of cells

Data from some cells should entirely be discarded due to the failure of the protocol in those cells. Multiple criteria to identify such problematic cells were proposed: *robustness to downsampling* [93], *fraction of read-pairs sequenced only once* [68] and *fraction of non-digested DNA* [69]. The most commonly used criterion is *cell coverage*, that is, the total number of detected contacts per cell [69]. For example, the cell coverage in sciHi-C follows the bimodal distribution, with low-coverage cells likely representing in-solution DNA noise [76]. Yet, another popular criterion is cumulative contacts properties, such as *cis-to-trans ratio* [68, 69, 76], defined as the ratio of the intrachromosomal contacts to the interchromosomal contacts. Typically, interchromosomal contacts in the chromatin occur with a lower probability than intrachromosomal ones, a phenomenon called *chromosome territoriality* [17]. Artifactual contacts are less likely to depend on the 3D distance between corresponding genomic positions and, thus, a deviating cis-to-trans ratio for a cell might signify excessive spurious ligation. Similar assumptions are used to filtrate the cells by *distance decay properties* of contacts [69] and *cross-species ligation frequency* [69, 76]. Another notion guiding the choice of high-quality cells is that scHi-C contacts tend to be found in clusters. Based on this observation, *GiniQC* measures the level of unevenness of inter-chromosomal scHi-C maps [40].

## Data structure

The scHi-C contact data are typically represented as a matrix, similar to the standard Hi-C [68]. Each cell in this matrix corresponds to a pair of genomic bins, and the value in a cell is the absolute number of interactions between these bins. A set of experiments is stored as a set of matrices, while specialised file formats exist to store matrices for a number of cells, such as scool [96]. Hypergraphs [99] and ‘topics’ [49] are representations for a set of cells used for specialised applications, such as prediction of contacts using machine learning [99] and data decomposition [49]. For special applications, scHi-C can be represented as a vector, for example, when scRNA-Seq methods are transferred to 2D data [37]. The 3D model is a popular representation, although it requires substantial preprocessing of the data and is not necessarily back-convertible to the set of initial contacts [68, 82, 86].

### Graph representation

**Figure 3:**
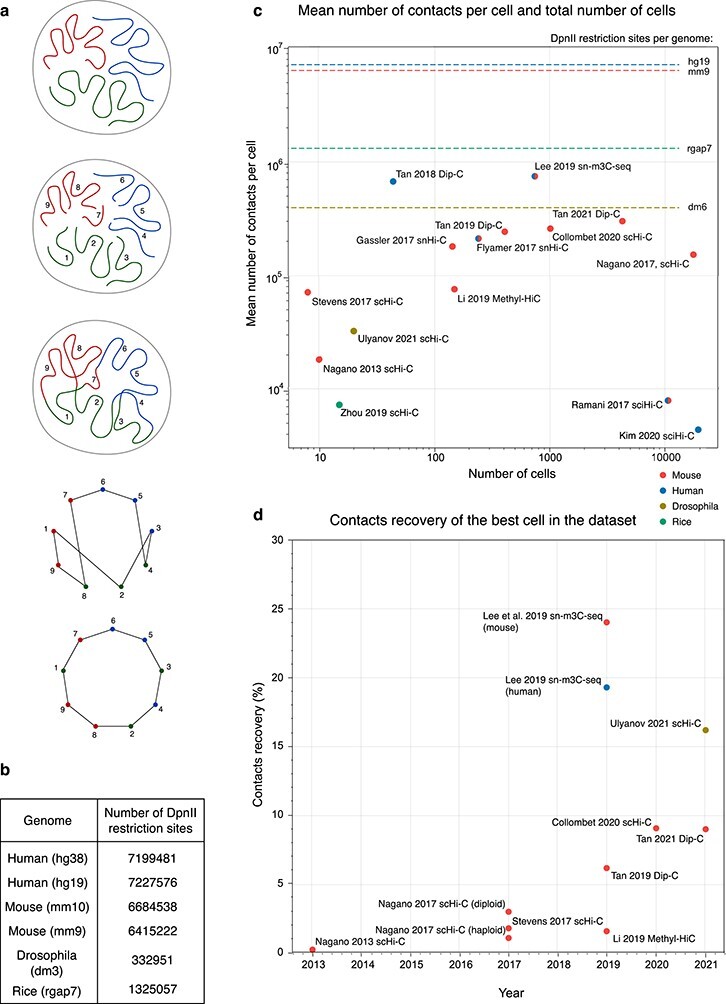
**A**. Illustrative upper bound estimation of the possible number of pairwise contacts per single cell. The theoretical genome has nine restriction fragments that form a circle graph after ideal ligation (nodes are restriction fragments with the valency of 2, edges denote ligation of their ends). **B**. Total numbers of DpnII restriction sites for the single copies of popular genomes. **C**. Descriptive statistics of published scHi-C studies. The lines represent the upper bounds for the possible number of contacts per single cell from (B). Colour indicate species. **D**. The best cells for some of the published scHi-C datasets as a function of the publication time. For C and D, we use the numbers reported in the supplementary materials of the original studies, when possible. For each study, we indicate the first author and the names of scHi-C techniques self-reported by the authors. For [49] and [76], the mean is calculated based on the median count per dataset. For [86], we used the cleaned contacts after removal of damaged cells. For [55], the calculated mean is based on the numbers reported for 741 cells in the supplementary table.

Graph representation [10, 100] is a popular representation that can be used to *upper bound for the number of pairwise contacts in scHi-C maps* [93] (Figure 3A). This upper bound can be defined for scHi-C but not bulk because a single cell with defined DNA content is used in the experiment. It depends on the number of restriction fragments that can potentially form contacts, which in each cell depends on the restriction site frequency, the organism’s genome size and the number of DNA copies in a particular cell type. For example, a single copy of the mouse genome mm10 [15] contains 6.6 million DpnII restriction sites (Figure 3B). In theory, if both ends of each restriction fragment were ligated to the ends of other restriction fragments and all ends are ligated, then the fragments form a circle graph. Thus, the number of contacts that could be detected would equal to the number of restriction fragments (Figure 3A). If two copies of the mouse genome are present (in a diploid cell), the number of possible contacts will be around 13.2 million. This number may be higher for cells during mitosis, S or G2 phase of the cell cycle, when the genomic content, and hence the number of restriction fragments, is completely or partially doubled. Although non-realistic to achieve in the working conditions of scHi-C, this number can serve as a theoretical upper bound to the possible number of pairwise contacts in a single nucleus. Notably, the largest number of contacts per cell obtained to date for mammals [57, 85] is already larger than the theoretical limit for the haploid genome of *Drosophila melanogaster* (Figure 3C), suggesting that the complete recovery of contacts of small genomes is possible with scHi-C.

The upper bound estimate can serve as a normalisation factor for contacts recovery in scHi-C studies (Figure 3D). The best standard scHi-C [93] has $17\%$ contacts recovery and the joint assay with methylation, sn-m3C-seq, almost reaches $25\%$ [55]. It is important to note that for an ideal scHi-C with $100\%$ recovery, we still cannot expect more than 2.4 interactions per 1 Kb of the genome (for haploid mm10 genome). This number is two orders of magnitude lower than bulk Hi-C (around 1700 contacts per 1Kb or genome in neural progenitor cells [7]). Thus, even if the theoretical limit is reached, scHi-C remains profoundly sparse and specialised software is required for its downstream analysis.

## Data analysis

There are two general approaches to the scHi-C data analysis, depending on the solution to the problem of low statistical power of scHi-C data sparsity. In the first one, every single cell is processed independently. It includes building its 3D model, data imputation, aggregation analysis and features calling. In the second approach, single-cell maps are analysed together, then grouped and pooled to produce pseudo-bulk Hi-C maps.

**Figure 4:**
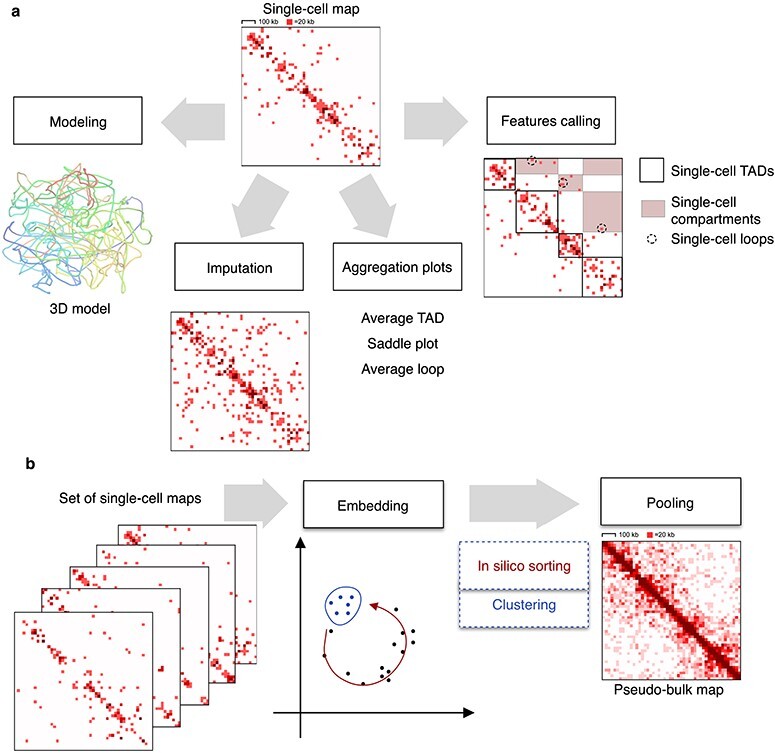
Approaches to studying a single scHi-C map (**A**) and a set of scHi-C maps (**B**). Single-cell Hi-C maps from [28] for the region chr1:9000000-1000000.

### Structure reconstruction

A typical approach for the 3D structure reconstruction is to build a *beads-on-string model* restrained by molecular dynamics with simulated annealing [68]. Each bead corresponds to a genomic bin of a given size (ranging from 10 Kb [93] to 1 Mb [69]), while each bond is either a polymer backbone or an observed scHi-C contact. The simulation starts from a random initial conformation, where the beads involved in observed scHi-C interactions might be overstretched. The beads connected by bonds are attracted to each other, forcing a rearrangement of the structure so that connected beads are located in close spatial proximity. Some bonds do not balance and remain overstretched; thus, they can be removed [82, 93] as potential experimental artefacts [53]. Other proposed solutions include Bayesian inference [11], recurrence plots [39] and lattice models [103]. All these methods remain data driven and do not account for the actual mechanisms of chromatin structure formation [43].

### Imputation of missing data

Due to contacts sparsity, applications of bulk Hi-C analysis tools to scHi-C are restricted [59]. To mitigate this effect, imputation techniques bring the numbers of scHi-C contacts closer to bulk [102]. Zhou *et al.* [100] populate the map with contacts generated by a random walk, making the scHi-C graph closer to a complete clique. Stevens *et al.* [82] and Ulianov *et al.* [93] use the maps imputed by polymer models. Notably, both TADs and compartments can also be readily assessed from model-imputed maps [82, 93], with TADs similar to those in original scHi-C data [93]. As a substantial breakthrough in scHi-C data imputation, inter-cellular patterns of contacts can be accounted for by the hypergraph neural network [99]. Some studies test the technical possibility to transfer dropout imputation algorithms for single-cell RNA-Seq, although lacking theoretical support [37].

### Contacts aggregation and features calling

**Figure 5:**
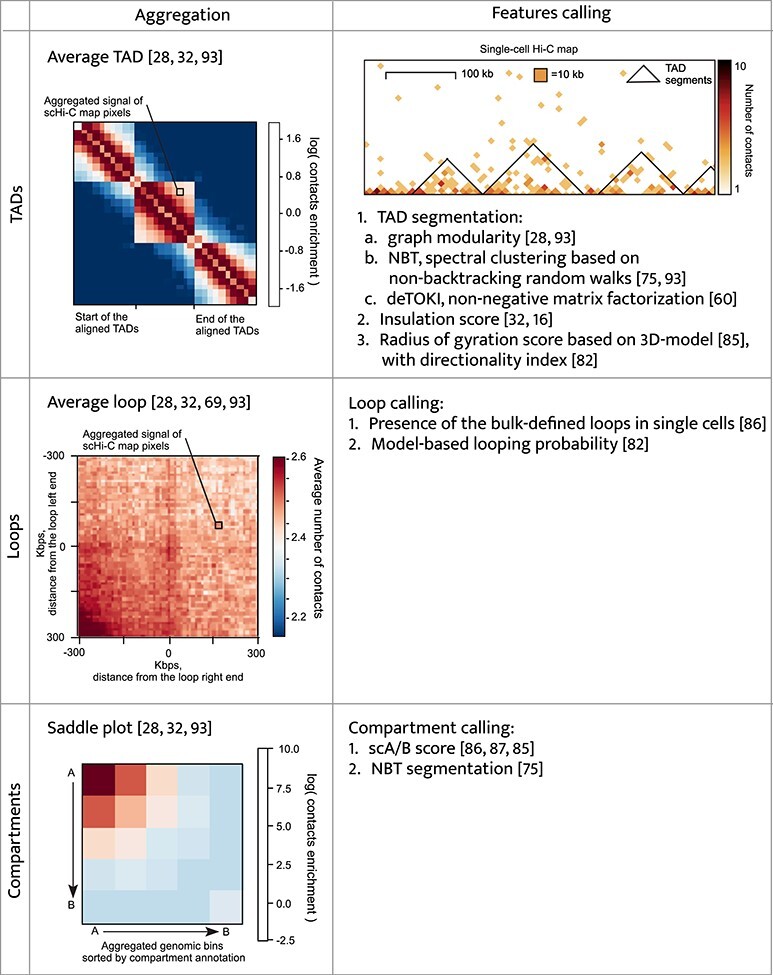
Comparison of aggregation of contacts and features calling for TADs, loops and compartments. All the examples are for the *Drosophila* scHi-C map of Cell 1 from [93]. Average TAD and saddle plot are for bulk TADs and compartments, while average loop is for the top 1000 regions with the highest content of RED chromatin state from [48].

Two approaches have been suggested to study TADs, loops and compartments in scHi-C maps, aggregation analysis and *features calling* (Figure 5). During *aggregation*, the statistics of contacts is accumulated over predefined regions of the genome (e.g. CTCF binding positions to assess loops; bulk TADs or bulk compartments). Aggregation confirms the presence of these chromatin features in individual cells [32], and there is specialised software for this purpose [29]. With *features calling*, the positions of individual loops [82], TADs [28, 60, 75, 93] and compartments [75, 86] are found directly in the scHi-C map, demanding high-quality scHi-C maps and providing insight into variability between individual cells. For example, the positions of TADs in individual cells demonstrated higher stability of TAD boundaries between individual cells of *Drosophila* than between mouse oocytes [93].

### scHi-C embedding

scHi-C data are multidimensional ($\sim N^2$ contacts measurements for N genomic regions) and can be projected into a space of lower dimension for visualisation, clustering and sorting. Typical visualisation is a scatter plot where each dot is a cell, and the axes correspond to some characteristics of the cells. The values on the axes can be derived from some additional measurement, such as the levels of the DNA replication marker geminin and DNA content from FACS [69] or the level of DNA methylation [55, 57]. Alternatively, the axes can represent some explicitly calculated interpretable characteristic of the scHi-C maps, such as the total number of contacts, the cis-to-trans ratio [16, 69] and the percentage of local/mitotic contacts [16, 69]. Tan *et al.* [86] characterise the 3D models instead, plotting the strength of the Rabl configuration, the centrality of telomeres, the number of interchromosomal neighbours, the average CpG content of the neighbours and the probability of cell-type-specific loops.

Finally, the axes might not readily correspond to any known biological characteristics—scHi-C maps can be transformed and subjected to *dimensionality reduction* by the *principal component analysis (PCA)* or other techniques (see Table 1 for comparison). For example, Ramani *et al.* [76] apply PCA to matrices of inter-chromosomal interactions and find that the first component explains a large fraction of the variance ($52.1\%$) and strongly correlates with the coverage. The combination of the first and second ($1.07\%$ explained variance) components distinguishes cell types. Nagano *et al.* [69] observe the cell cycle-dependent embedding of scHi-C by calculating the pairwise symmetric Kullback–Liebler divergence on vectors of distance decays and subsequent spectral embedding. Collombet *et al.* [16] apply *uniform manifold approximation and projection (UMAP)* to vectors of TAD contact profiles; Li *et al.* [60] perform PCA on pairwise similarities of TAD profiles; Tan *et al.* [87] calculate the compartment score profiles for each cell, take 20 principal components and then visualise it with *t-distributed stochastic neighbour embedding (t-SNE)*. One of the most generalised approaches is HiCRep [100], which calculates a similarity matrix between each pair of individual cells, taking the *stratum-adjusted correlation coefficient (SCC) * measure of similarity. HiCRep with *subsequent multidimensional scaling (MDS)* has proved to be one of the best approaches to study embedded scHi-C datasets [65]. In this approach, Zhou *et al.* [100] propose to impute potential dropouts before the embedding to increase the cluster separation. The imputation was further supplemented it with scRNA-Seq dropout correction methods [37] (but see the discussion above).

**Table 1:** Summary of major scHi-C embedding techniques

Family of embedding methods	Linearity	Primary reference	Special scHi-C pre-processing	Special measure of similarity/difference between cells	Explicit usage of contacts co-occurrence patterns
PCA	Linear	[100]	Raw binned matrix	–	No
		[76]	Interchromosomal interactions profile	–	No
		[60]	TAD profile	–	No
		[87]	Compartment score profile	–	No
t-SNE	Non-linear	[85]	20 PCs of compartment score profiles	–	No
Spectral embedding	Non-linear	[69]	Distance decays	Symmetric KL	No
MDS	Non-linear	[65]	Distance decay	Jensen–Shannon divergence	No
		[100]	scHi-C binned matrix after smoothing and random-walk imputation	SCC	No
UMAP	Non-linear	[16]	TAD contact profiles	–	No
		[49]	Cell-topic matrix after LDA	–	Yes
		[99]	Hypergraph embedding	–	Yes

MDS indicates multidimensional scaling; SCC, stratum-adjusted correlation coefficient; UMAP, uniform manifold approximation and projection.

An alternative, scHiCExplorer [96], implements an approximate nearest neighbour method with a local sensitive hash function, MinHash. Finally, some approaches suggest using the co-occurrence of contacts in individual cells to base the embedding on meaningful single-cell patterns. For example, Kim *et al.* [49] applied *latent Dirichlet allocation* to factorise the scHi-C dataset into a set of documents, words and topics, and Zhang *et al.* [99] used a *hyper-graph neural network*. In all these studies, the axes created by *in silico* approaches are rarely interpreted, and it might be of interest to correlate them with various scHi-C characteristics such as the contact coverage, distance decay, strength of TADs, loops and compartments.

A more exotic approach is to describe scHi-C space in terms of topological data analysis [10]. Finally, joint assays of the methylome and interactome [55, 57] allow for independent embeddings of scHi-C and single-cell methylation patterns and subsequent comparison of resulting embeddings.

To date, no comprehensive studies on embedding all existing scHi-C datasets have been published. Moreover, there have been no attempts to embed datasets originating from different species, although scHi-C data for human [28, 49, 76, 86], mouse [16, 68, 69, 76, 82, 85, 87], *Drosophila* [93] and rice [101] are available. This might identify species-specific patterns in genomic interactions and their variability.

While both linear and non-linear embeddings of scHi-C have been proposed, advanced *manifold learning* techniques are yet to be developed for scHi-C, analogous to the outbreak of embedding methods for single-cell RNA-Seq data (reviewed in [67]). At that, multiple, diverse formalisations of scHi-C as matrices, graphs and vectors allow for a broad field of embedding techniques to be studied on these datasets.

### *In silico* sorting, clustering and pooling

Based on the position in the embedding space, scHi-C data can be *in silico* sorted [69] or clustered [85]. Nagano *et al.* [69] observed the ordering of the cells by the position in the cell cycle, while Tan *et al.* [85] derived subtypes of mouse brain cells using k-means. Collombet *et al.* [16] relied on outliers in the embedding space to filter out cells undergoing mitosis and retain only interphase embryonic cells.

Specialised approaches, including the ones based on machine learning, have been designed for scHi-C data clustering. Typically, these applications require embedding (see below). The quality of clustering is tested on datasets with known ground truth (e.g. types of pronuclei in the mouse zygote [28] or types of cells forming the dataset [76]). Each cluster, or group of cells, is assigned with a particular cell type and the quality is usually assessed by normalised mutual information [62] or adjusted rand score [62, 100].

The resulting groups of cells can be pooled by simple summation of single-cell Hi-C maps, resulting in *ensemble*, or *pseudo-bulk*, Hi-C and analysed as typical bulk Hi-C [16, 69, 85]. Pseudo-bulk scHi-C maps are a powerful technique for detection of cell-type specific differences in the chromatin architecture. For example, pseudo-bulk mitotic cells lack the TAD and compartment structure [69], while subtypes of brain cells have differences in regions of cell-type specific genes [85].

The long-studied field is the reverse of the pooling, namely deconvolution of bulk interaction maps into a set on single cells [46]. Such approaches aim to construct a population of genome structures with a total set of genomic interactions approximating (or equal to) a set observed in a population of nuclei. Several advanced techniques including machine learning have been suggested, such as *maximum likelihood* [89], *Bayesian inference* [12], *fractal Monte Carlo weight enrichment with Bayesian deconvolution* [74], *Monte Carlo with bag of little bootstraps* for the generation of bootstrap structures [83] and, most recently, stochastic embedding [36]. However, these approaches are limited by the number of models that approximate bulk datasets (up to several tens of thousands), although around 5–10 million structures contribute to the typical bulk Hi-C map. Nevertheless, it might be interesting to demonstrate the reversibility of the pooling of a low number of single-cell maps by applying some of these methods to pseudo-bulk datasets. Guarnera *et al.* [36] assessed the variability of polymers after deconvolution, which might be interesting to compare with results obtained from embeddings of real scHi-C.

### Design of scHi-C controls

Due to the complex nature of scHi-C data, a good practice is to design scHi-C controls to validate the hypotheses. These include *sampled, shuffled* or *de novogenerated randomised scHi-C maps*, which typically have the same number of contacts as real cells. *Sampled maps* are populated by contacts randomly selected from bulk [93] or ensemble [68, 76] datasets. However, it creates maps less sparse and heterogeneous than real scHi-C maps [100]. Thus, an effective number of sampled contacts can be increased or additional artificial noise can be introduced [100]. *Shuffled maps* are single-cell maps with randomly permuted pairs of contacts [68]. This procedure retains coverage by contacts but removes any information on the spatial structure, including distance decay. Sampling and shuffling can be combined together: bulk Hi-C maps first randomised, preserving the coverage and distance decay, and then sampled [69]. *De novogenerative models* do not rely directly on the observed contact maps while preserving the meaningful properties of scHi-C maps. For example, thresholding the distance between genomic regions in polymer models [93] produces control maps with meaningful distance decays. A more advanced alternative, stepwise generation of single-cell Hi-C-like maps, preserves both distance decay and observed coverage by contacts [93].

Controls like this allow differentiating the technical and biological properties of the single-cell contact maps for features calling (such as TADs) and aggregation analysis [93]. They provided the baseline for assessing the general quality of modelling by the number of violated constraints [68]. Further, they demonstrated that scHi-C maps are non-random [82] and chromatin features of the modelled cells are similar to that of the real cells [69, 82, 93]. Yet another important observation is that real scHi-C data are more variable and sparse than bulk subsamples [100]. Although randomised scHi-C control is a powerful method, it is sporadically used in scHi-C studies. This will improve with the development of specialised tools for this task and the emergence of theoretical studies on the statistical properties of single-cell contacts.

## Outlook and challenges

Single-cell Hi-C is a young and rapidly developing field in chromatin biology. Due to its extreme data sparsity and complicated experimental protocol, the quality of the datasets has been a limiting factor. However, with the emergence of simplified and cheaper protocols [76, 86], we anticipate continued growth of both coverage of scHi-C and number of cells analysed, leading to improved data resolution and statistical reliability of the biological results. This will also stimulate the development of new data processing and analysis methods. However, as we demonstrated here, scHi-C data have a natural upper bound for the possible number of recovered single-cell interactions; thus, data sparsity will remain a challenge for the field.

Despite the substantial efforts to work with sparse data, the computational analysis of scHi-C has not reached maturity yet. For example, a recent re-analysis of datasets from three studies demonstrated that inappropriate contacts mapping may result in the accumulation of experimental artefacts and overestimation of the number of recovered contacts [93]. However, if the data from multiple studies were processed uniformly, it demonstrated that TAD boundaries in *Drosophila* are more conserved than in mouse. Similar comparative analysis of scHi-C results will further shed light on reproducible chromatin features in individual cells in an unbiased way.

Machine learning has a growing impact on our understanding of biological systems (reviewed in [27, 63]) and 3D genomics [4, 30, 77, 79, 94, 95]. For single-cell chromatin research, imputation and embedding are already driven by neural networks [99] and other advanced machine learning methods will emerge. Importantly, features calling from single-cell data will be improved.

Next, an important direction is improving structural reconstruction approaches. To date, scHi-C structure reconstruction does not account for a specific mechanism of structure formation. Alternative *de novo* modelling assumes the particular mechanism but does not incorporate scHi-C contacts [28, 30]. These approaches can be, in theory, united to open intriguing perspectives. For example, can we simulate loop extrusion [31] that will produce the contact maps similar to those observed in scHi-C? Can we infer the cohesin loading sites in individual cells based on observed contacts? Finally, can we differentiate the cohesin-dependent contacts in single cells from compartmental ones [32] and study them independently?

These challenges are not the only ones that will require computational solutions. An important direction will be the design of new assays, as well as tools for their data processing. For example, currently, restriction enzymes digest chromatin into relatively large restriction fragments, which dictates the strict upper bound for the total number of pairwise contacts recoverable from a single cell. If micrococcal nuclease is used instead, it will allow for up to 15 million contacts of individual nucleosomes in the haploid human genome [1], increasing the theoretical upper bound at least twice.

Joint assays, other than Methyl-3C and sc-me3C, will unravel the interplay of chromatin architecture with other cellular mechanisms. For example, measuring single-cell lamina-associating domains (LADs) alongside scHi-C will shed light on the lamina association of individual TADs. Indeed, bulk TADs do not entirely correspond to either bulk [91] and single-cell LADs [50]. However, it is possible that single-cell TADs are elementary units of interaction with lamina if there is a one-to-one correspondence between TADs and LADs observed in the same cell. Next, measuring chromatin openness and/or transcriptional activity will accelerate the research on interplay and causality between regulation, chromatin folding and gene expression [24]. On the computational side, having more than one type of measurement in single cells is a unique opportunity to develop joint embedding [56] methods, which use both interaction graphs and single-cell features to create meaningful low-dimensionality representation. Also, having several types of measurements will help to develop and benchmark standard scHi-C embedding techniques.

Single-cell RNA-DNA contacts will help distinguish RNA-mediated interactions from the rest and depict the single-cell pattern of regulatory RNA functioning. However, the resolution of bulk RNA-DNA interaction capture techniques is relatively low [3, 33, 61, 81], which will remain a major impediment for single-cell RNA-DNA interactions as well.

Currently, scHi-C requires vast sequencing with relatively low meaningful output (e.g. Ramani *et al.* [76] sequenced over 170 mln reads per dataset on average, only $11\%$ of them resulting in unique contacts). However, studying biological mechanisms of chromatin compaction and regulation frequently requires engineering and targeting of individual regions of the genome limited in size. Thus, it might be beneficial to develop single-cell Hi-C with enrichment for targets. Target enrichment for a genomic region is already well developed for bulk chromosome capture approaches [20, 25, 35]. Adaptation of these approaches for the single-cell level will allow for specific enrichment of single-cell interactions of regulatory regions that might undergo the specific architectural changes in a cell population.

As both wet-lab and computational scHi-C methods improve, it will lead to breakthroughs in understanding biological systems currently restricted by bulk Hi-C. For example, chromatin transitions during mouse embryogenesis were studied by low-input Hi-C [26, 47], which accommodates the limited number of embryos available but does not distinguish individual cells. Starting from the zygote and up until the gastrulation (stage E7.5), chromatin features gradually emerge. At stage E7.5, the embryo has approximately 15000 cells, some differentiated into progenitors of diverse tissues and organs [90]. Their variability can be recovered only by scHi-C. Indeed, scHi-C demonstrated cell- and allele-specific patterns of chromosomes folding in mouse embryos but only up to a much earlier stage of 64 cells [16, 28, 32]. Given the fact that existing scHi-C assay several tens of thousands of cells [49], a whole-embryo single-cell chromatin structure study is a realistic short-term goal. This opens an intriguing perspective to answer fundamental questions about chromatin dynamics in development. What paths do chromosomes follow in individual nuclei during tissue differentiation and organogenesis? Can we track the lineages of cells based on their chromatin, as we do for single-cell transcription [80]? Finally, what are the rules governing chromatin transitions in individual cells during the development of other species studied by bulk Hi-C, including human [14], *Xenopus tropicalis* [71], Medaka fish [70], *Danio rerio* [45] and *Drosophila melanogaster* [41]?

Next, scHi-C will uncover the diversity of chromatin architecture within cancer cell, contributing to the clonal analysis of solid and liquid tumours currently done with genomic and transcriptomic methods. Finally, single-cell atlases of chromatin architecture for cell types of different organs will expand our knowledge on chromatin structural diversity. Their proper association with single-cell atlases of transcription [38] and chromatin openness [18, 98] will unravel the interplay between epigenetics, chromatin structure and gene expression.

**Key Points**

1.
$\bullet $ Single-cell Hi-C is a powerful and rapidly developing technology to study chromatin architecture, with computational analysis playing a crucial role in extracting biological meaning from its sparse readouts.
2.
$\bullet $ The number of scHi-C pairwise genomic contacts is limited by the number of genomic fragments in the nucleus requiring special approaches for sparse interactome data analysis, including structure reconstruction, imputation of interactions, aggregation of contacts and feature calling for a single map and embedding, sorting, clustering and pooling for a set of maps.
3.
$\bullet $ We anticipate improvements in scHi-C data quality and computational analysis to lead to the expansion of scHi-C applications, eventually resulting in breakthroughs in our understanding of cell function comparable with those achieved by scRNA-seq and scATAC-seq.

## Notes

**Aleksandra Galitsyna** is a PhD student in Prof. Gelfand’s group at Skolkovo Institute of Science and Technology. Her scientific agenda includes various topic in the broad field of chromatin research focused on biology and bioinformatics of single cells.

**Mikhail Gelfand** is a bioinformatician, professor at the Center of Life Sciences and vice president for biomedical research at the Skolkovo Institute of Science and Technology, Moscow, Russia. His scientific interests include a broad range of problems, ranging from bacterial genomics and evolution to transcriptomics, splicing and mRNA editing in eukaryotes, with chromatin structural analysis being one of them.
